# Supplementary material for: The Small RNA Universe of Capitella teleta
Source: Front Mol Biosci. 2022 Feb 25;9:802814. doi: 10.3389/fmolb.2022.802814 (PMC8915122; doi:10.3389/fmolb.2022.802814)
Supplement: Supplementary file 1 [file DataSheet1.ZIP › Supplement/known/CAPTEscaffold_194_12905.pdf]

Provisional ID	:	CAPTEscaffold_194_12905
Score total	:	17302.1
Score for star read(s)	:	3.9
Score for read counts	:	17291.4
Score for mfe	:	2.2
Score for randfold	:	1.6
Score for cons. seed	:	3
Total read count	:	33928
Mature read count	:	28926
Loop read count	:	0
Star read count	:	5002

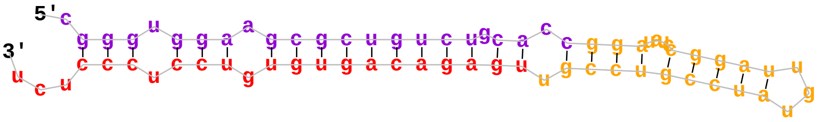

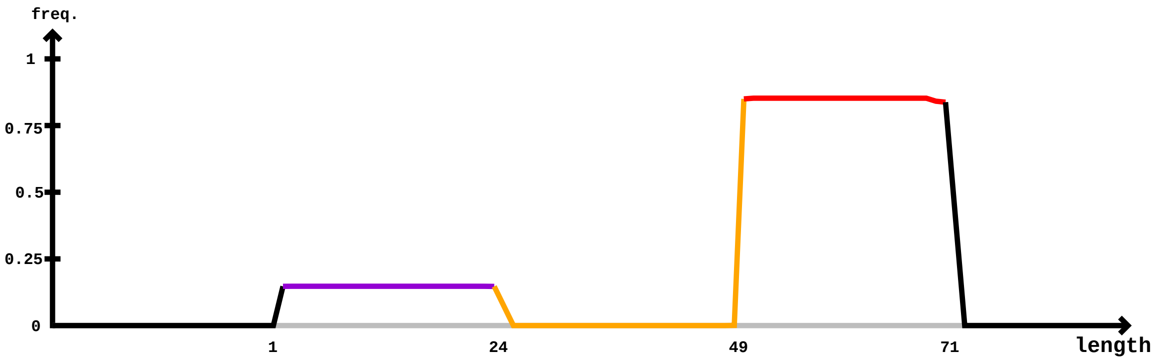

Star **Mature**

```
5'- auuucacugagcgguugugcaucggguggaagcgcugucugcaccggauauccggauuguauccguccguugagacaguguguccucccucuggacgccguucacucuca  -3'   obs
    auuucacugagcgguugugcaucggguggaagcgcugucugcaccggauauccggauuguauccguccguugagacaguguguccucccucuggacgccguucacucuca         exp
    auuucacugagcgguugugcaucggguggaagcgcugucugcaccggauauccggauuguauccguccguugagacaguguguccucccucuggacgccguucacucuca         known
    .......((((((((.((.((..(((.(((.(((((((((.((.((((....(((((...))))))))).))))))))))).))).)))..)).))))))))))......   reads   mm   sample
    .....................Gcggguggaagcgcugucugcacc.................................................................   1       1    seq
    ......................cggguggaagcgcugucugca...................................................................   4       0    seq
    ......................cggguggaagcgcugucugcU...................................................................   1       1    seq
    ......................cggguggaagcgcugucugcac..................................................................   15      0    seq
    ......................cggguggaagcgcugucuAcacc.................................................................   3       1    seq
    ......................Aggguggaagcgcugucugcacc.................................................................   4       1    seq
    ......................Nggguggaagcgcugucugcacc.................................................................   1       1    seq
    ......................cggguUgaagcgcugucugcacc.................................................................   1       1    seq
    ......................cggguggaagcgcugucCgcacc.................................................................   2       1    seq
    ......................cggguAgaagcgcugucugcacc.................................................................   8       1    seq
    ......................cggguggaGgcgcugucugcacc.................................................................   1       1    seq
    ......................cggguggaagcgcuguGugcacc.................................................................   2       1    seq
    ......................Gggguggaagcgcugucugcacc.................................................................   1       1    seq
    ......................cggguggaagcgcugucuUcacc.................................................................   1       1    seq
    ......................cggguggaagcgcugucugcacc.................................................................   4854    0    seq
    ......................Uggguggaagcgcugucugcacc.................................................................   2       1    seq
    ......................cggguggaagcgcuguAugcacc.................................................................   2       1    seq
    ......................cggguggaagcgcugucugAacc.................................................................   2       1    seq
    ......................cgggugCaagcgcugucugcacc.................................................................   1       1    seq
    ......................cggguggaagcAcugucugcacc.................................................................   4       1    seq
    ......................cggguggGagcgcugucugcacc.................................................................   1       1    seq
    ......................cggguggaagAgcugucugcacc.................................................................   1       1    seq
    ......................cggguggaagcgUugucugcacc.................................................................   1       1    seq
    ......................cgggAggaagcgcugucugcacc.................................................................   4       1    seq
    ......................cggguggaagcgcugucugcacU.................................................................   10      1    seq
    ......................cggguggaagcgcugAcugcacc.................................................................   3       1    seq
    ......................cggguggUagcgcugucugcacc.................................................................   1       1    seq
    ......................cgggGggaagcgcugucugcacc.................................................................   1       1    seq
    ......................cggguggaagcgcuCucugcacc.................................................................   1       1    seq
    ......................cAgguggaagcgcugucugcacc.................................................................   12      1    seq
    ......................cggguggaagcgcugucugcGcc.................................................................   2       1    seq
    ......................cggguggaagcgcGgucugcacc.................................................................   1       1    seq
```

```
                      Star                                              Mature
auuucacugagcgguugugcaucggguggaagcgcugucugcaccggauauccggauuguauccguccguugagacaguguguccucccucuggacgccguucacucuca
......................cggguggaagcgcugucugcacA.................................................................   1      1   seq
......................cgAguggaagcgcugucugcacc.................................................................   6      1   seq
......................cggguggaagcgcuAucugcacc.................................................................   1      1   seq
......................cggguggaagcgcugucugcaccU................................................................   35     1   seq
.......................ggguggaagcgcugucugcacc.................................................................   11     0   seq
.....................................................................uugagacaguguguccucccuc...................   2      0   seq
.....................................................................uugagacaguguguccucccucu..................   10     0   seq
.....................................................................Cugagacaguguguccucccucu..................   7      1   seq
.....................................................................uugagacaguguguccucccucuU.................   2      1   seq
......................................................................ugagacaguguguccuccc.....................   2      0   seq
......................................................................ugaAacaguguguccucccu....................   2      1   seq
......................................................................ugagacagugGguccucccu....................   2      1   seq
......................................................................ugagacaguguguccucccC....................   1      1   seq
......................................................................ugagacaguguguccuccAu....................   1      1   seq
......................................................................ugagacaguguguccucccu....................   365    0   seq
......................................................................ugagacaguguguccuAccu....................   1      1   seq
......................................................................Agagacaguguguccucccu....................   1      1   seq
......................................................................ugagGcaguguguccucccu....................   2      1   seq
......................................................................ugaUacaguguguccucccu....................   1      1   seq
......................................................................ugagacaguUuguccucccu....................   1      1   seq
......................................................................ugagacaguguguccucccA....................   1      1   seq
......................................................................ugagacaguguguccucccuc...................   120    0   seq
......................................................................ugagacaguguguccucccuU...................   1      1   seq
......................................................................Agagacaguguguccucccuc...................   2      1   seq
......................................................................ugagacaguguguccGcccucu..................   3      1   seq
......................................................................ugagacaguguguccucccGcu..................   2      1   seq
......................................................................uUagacaguguguccucccucu..................   4      1   seq
......................................................................ugagacaguguguccucccuAu..................   1      1   seq
......................................................................ugagacaguguguccuGccucu..................   4      1   seq
......................................................................ugaCacaguguguccucccucu..................   3      1   seq
......................................................................ugagacaguguguccucccCcu..................   3      1   seq
......................................................................ugUgacaguguguccucccucu..................   3      1   seq
......................................................................ugagaUaguguguccucccucu..................   5      1   seq
......................................................................ugagacaguguguccucAcucu..................   9      1   seq
......................................................................ugagacaguguguccucccucA..................   12     1   seq
......................................................................ugagacaguUuguccucccucu..................   1      1   seq
......................................................................ugagCcaguguguccucccucu..................   1      1   seq
......................................................................ugagacagugugucUucccucu..................   24     1   seq
......................................................................Ngagacaguguguccucccucu..................   9      1   seq
......................................................................ugagacaguguCuccucccucu..................   2      1   seq
......................................................................ugagacagugGguccucccucu..................   4      1   seq
......................................................................ugagacaguNuguccucccucu..................   1      1   seq
......................................................................ugagacaguguguccuAccucu..................   5      1   seq
......................................................................Ggagacaguguguccucccucu..................   10     1   seq
......................................................................ugagacaguguguccuccAucu..................   8      1   seq
......................................................................ugagacagugugu Acucccucu..................   3      1   seq
......................................................................ugagacagAguguccucccucu..................   17     1   seq
......................................................................ugagacaguguAuccucccucu..................   3      1   seq
......................................................................ugagacagugugAccucccucu..................   7      1   seq
......................................................................ugaAacaguguguccucccucu..................   4      1   seq
......................................................................ugGgacaguguguccucccucu..................   8      1   seq
......................................................................ugagacaCuguguccucccucu..................   3      1   seq
......................................................................uCagacaguguguccucccucu..................   1      1   seq
......................................................................ugagacaguguguccucccucG..................   31     1   seq
......................................................................ugagacaguguguccucGcucu..................   1      1   seq
......................................................................ugagacagGguguccucccucu..................   3      1   seq
......................................................................ugagacaguguguccucccuUu..................   4      1   seq
......................................................................ugagacaguCuguccucccucu..................   3      1   seq
......................................................................ugagacaguguguccCcccucu..................   3      1   seq
......................................................................ugagacaguguguccucccucu..................   27341  0   seq
......................................................................Cgagacaguguguccucccucu..................   6      1   seq
......................................................................ugagacaUuguguccucccucu..................   1      1   seq
......................................................................ugagacagugugCccucccucu..................   7      1   seq
......................................................................ugagacaguguguccucUcucu..................   35     1   seq
......................................................................ugagacagugCguccucccucu..................   2      1   seq
......................................................................ugCgacaguguguccucccucu..................   1      1   seq
......................................................................ugagacagugugucAucccucu..................   12     1   seq
......................................................................ugagacaguguguccuccUucu..................   18     1   seq
......................................................................ugagacaguAuguccucccucu..................   4      1   seq
......................................................................ugagacaguguguccucccucC..................   7      1   seq
```

Star **Mature**

auuucacugagcgguugugcaucggguggaagcgcugucugcaccggauauccggauuguauccguccguugagacaguguguccucccucuggacgccguucacucuca			
....ugagGcaguguguccucccucu..................	7	1	seq
....ugagacaguguguccuccGucu..................	1	1	seq
....ugagacaguguguUcucccucu..................	7	1	seq
....ugagacaguguguccuUccucu..................	4	1	seq
....ugagacagugAguccucccucu..................	14	1	seq
....ugagaGaguguguccucccucu..................	2	1	seq
....ugagaAaguguguccucccucu..................	7	1	seq
....ugagacCguguguccucccucu..................	1	1	seq
....ugagUcaguguguccucccucu..................	1	1	seq
....Agagacaguguguccucccucu..................	100	1	seq
....ugagacaguguguccAcccucu..................	13	1	seq
....uAagacaguguguccucccucu..................	43	1	seq
....ugagacGguguguccucccucu..................	4	1	seq
....ugagacagugugucGucccucu..................	3	1	seq
....ugagacaguguguccucccucuA.................	167	1	seq
....ugagacaguguguccucccucuU.................	300	1	seq
....gagacaguguguccucccu....................	2	0	seq
....gagacaguguguccucccucu..................	82	0	seq
....gagacaguguguccucccuAu..................	1	1	seq
....Uagacaguguguccucccucu..................	1	1	seq
....gagacagugugAccucccucu..................	2	1	seq
....gagacaguguguccucccucuA.................	1	1	seq
